# Supplementary material for: A miR-9-5p/FOXO1/CPEB3 Feed-Forward Loop Drives the Progression of Hepatocellular Carcinoma
Source: Cells. 2022 Jul 5;11(13):2116. doi: 10.3390/cells11132116 (PMC9265408; doi:10.3390/cells11132116)
Supplement: Supplementary file 1 [file cells-11-02116-s001.zip › HCC-FFL-Supplementary Materials-revised.pdf]

Supplementary Materials

1. Supplementary Methods

1.1. High-throughput sequencing data

Samples from *FOXO1* overexpressed HepG2 cells were subjected to high-throughput sequencing: RNA-Seq (PE150) via Illumina NovaSeq6000 platform by HaploX Genomics Center (Jiangxi, China). We obtained 52-62 million clean reads for RNA per sample (Table S1). RNA-Seq reads were mapped to Ensembl v87 (GRCH38) and processed by the HISAT2-StringTie-ballgown pipeline. We used Fragments Per Kilobase per Million (FPKM) to calculate gene expression levels. Basic statistics of sequencing data were presented in Table S1. Analyses of DNA methylation and copy number variation were performed by GSCALite.

1.2. Luciferase reporter gene assay

Luciferase reporter gene assays were performed to validate CPEB3 as a direct target of miR-9-5p and FOXO1. For miR-9-5p targeting CPEB3, the gene segment of *CPEB3* 3' UTR was synthesized and ligated into a SpeI/HindIII digested pMIR-Reporter plasmid. The sequence of wild-type (WT) CPEB3 was taken as a reference, and the complementary sequence of the miRNA seed sequence was mutated. Two binding sites on *CPEB3* 3' UTR (*CPEB3* transcript variant 2, NCBI: NM_001178137.1) for miRNA-9-5p were predicted by TargetScan as shown in Figure 3D, which were named as CPEB3−1 (position 437-443 of *CPEB3* 3' UTR) and CPEB3-2 (position 1367-1373 of *CPEB3* 3' UTR), respectively. CPEB3−1 wild-type (WT) sequence: 5′-CCUAGGACAAGACUUACCAAAGG; CPEB3−1 mutant (MUT) sequence: 5′-CCUAGGACAAGACUUUGGUUUCG. CPEB3−2 (WT) sequence: 5′-GACCUUGUCCAGAAAACCAAAGA; CPEB3−2 mutant (MUT) sequence: 5′-GACCUUGUCCAGAAAUGGUUUCA. By using T4 DNA ligase, after digestion by restriction endonucleases, the target fragment was inserted into a pMIR-Reporter plasmid. The HEK-293T cells were transfected with the CPEB3−1 WT and CPEB3−2 WT plasmid or CPEB3−1 MUT and CPEB3−2 MUT plasmid and miR-9-5p mimics or negative control sequences. After 48 hours, cells were collected and lysed. Luciferase activity was measured using the luciferase assay kit (U6885-01).

For FOXO1 targeting CPEB3, the HepG2 cells were transfected with reporter plasmids together with PCDNA3.1 as an internal control. Expression vectors for *FOXO1* were kindly provided by Prof. Lu Gan. Plasmids (pGL3-Basic) for wild type and three binding sites of promoter of the *CPEB3* (site1: GCAACCACAAACATCATGAA of promoter of *CPEB3* isoform 1; site2: GTTTTTGTTT of promoter of *CPEB3* isoform 2; site3: GTAACACTAAACATGTACAA of promoter of *CPEB3* isoform 2) and the luciferase reporter construct, 3xIRS, which contains three copies of the FOXO1 response element in the promoter of the *CPEB3* gene. Cells were harvested 48 h post-transfection and renilla luciferase activity was normalized to firefly activity shown as relative luciferase activity by using a dual luciferase reporter assay system (Promega, Madison, WI, USA).

1.3. Chromatin Immunoprecipitation and Sequencing

Chromatin immunoprecipitation assays were performed as previously described. Briefly, 10 million cells were washed twice in cold PBS buffer and cross-linked with 1% formaldehyde (f8775, Sigma) for 10 minutes at room temperature and then quenched by addition of glycine to 125 mM. Afterward, samples were lysed and chromatins were obtained on ice. Chromatins were sonicated to get soluble sheared chromatins (average DNA length of 200–500 bp). 20 μl chromatin was stored at –20°C as input DNA, and 100 μl chromatin was used for immunoprecipitation by Anti-FOXO1A antibodies (ab39670, abcam). 10 μg of antibody was

used in the immunoprecipitation reactions at 4 °C overnight. 30 μl of protein beads were added and the samples were further incubated for 3 h. The beads were next washed once with 20 mM tris (hydroxymethyl) aminomethane hydrochloride (Tris-HCl) (pH 8.1), 50 mM NaCl, 2 mM EDTA, 1% Triton X-100, 0.1% SDS; twice with 10 mM Tris-HCl (pH 8.1), 250 mM LiCl, 1 mM EDTA, 1% NP-40, 1% deoxycholic acid; and twice with TE buffer 1× (10 mM Tris-HCl and 1 mM EDTA at pH 7.5). Bound material was then eluted from the beads in 300 μl of elution buffer (100 mM NaHCO3, 1% SDS), treated first with RNase A (final concentration 8 μg/mL) for 6 h at 65°C and then with proteinase K (final concentration 345 μg/mL) overnight at 45°C.

Immunoprecipitated DNA was used to construct sequencing libraries following the protocol provided by the I NEXTFLEX® ChIP-Seq Library Prep Kit for Illumina® Sequencing (NOVA-514120, Bioo Scientific) and sequenced on Illumina Xten with PE 150 method. Trimmomatic (version 0.38) was used to filter out low-quality reads. Clean reads were mapped to the Ensembl v87 (GRCH38) genome by Bwa (version 0.7.15), allowing up to two mismatches. Samtools (version 1.3.1) was used to remove potential PCR duplicates, and MACS2 software (version 2.1.1.20160309) was used to call TF binding peaks by default parameters (band width, 300 bp; model fold, 5, 50; P value, 0.01, a range for calculating regional lambda = 1000 and 10000 bps). The target prediction from high-confident motifs based on the peaks was performed according to the method from hTFtarget. Bedg files produced by MACS2 software were used for data visualization by UCSC (http://genome.ucsc.edu/).

1.4. Real-time qPCR analysis

Total RNA was isolated by TRIzol™ reagent (Life Technologies, Carlsbad, CA, USA) according to the instructions. Real-time quantitative PCR (qPCR) was performed using HiScript II One Step qRT-PCR SYBR Green Kit (Vazyme, Nanjin, China). The relative expression of mRNAs was normalized to human glyceraldehyde 3-phosphate dehydrogenase (GAPDH). Sequences of all the primers for real-time qPCR were listed in Table S3. All primers were designed by Primer Premier 6, and synthesized by TSINGKE (Wuhan, China). The expression of miRNA was detected by All-in-One™ miRNA qRT-PCR Detection kit (GeneCopoeia™, Guangzhou, China) according to the manufacturer's instructions. All miRNA expression was normalized to hsa-U6.

1.5. Western blotting assay

Cells and tumor tissues were lysed with RIPA Lysis buffer (Beyotime, China), protein concentrations were determined by the BCA kit (Beyotime, China). Each sample was separated by 8-12% SDS-PAGE, blocked with 5% BSA for 2 h at room temperature and incubated with primary antibodies to FOXO1 (Abcam, USA), CPEB3 (Abclonal, China), GAPDH (Abclonal, China), overnight at 4℃. Primary antibodies were detected with goat anti-Rabbit IgG-HRP or anti-Mouse IgG-HRP (Santa Cruz, TX, USA). The blots were developed using Super Signal West Pico chemiluminescent substrate (Millipore, MA, USA).

2. **Supplementary Figures and Tables**

2.1. Supplementary Figures

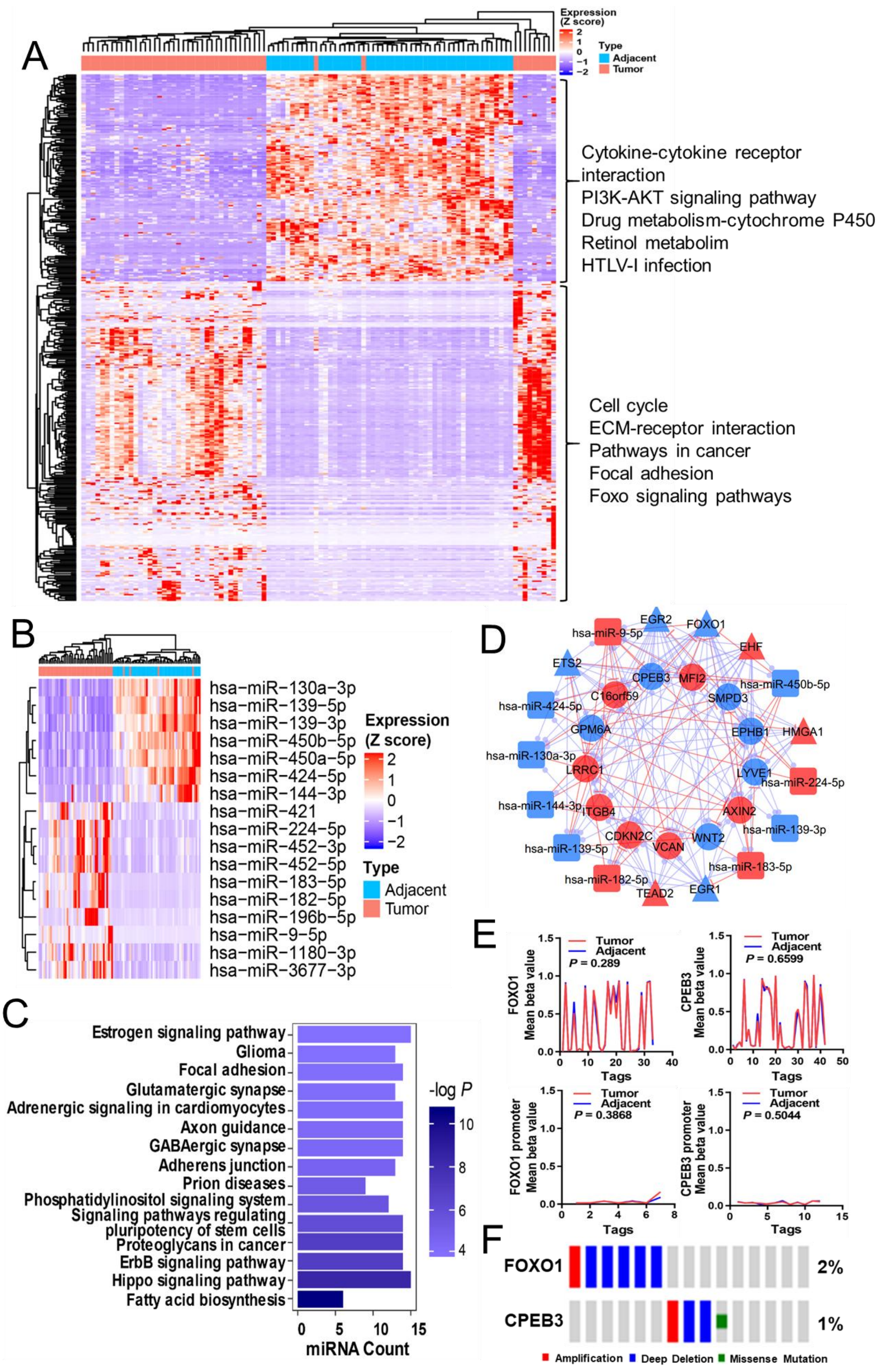

Figure S1. Functional analysis and miRNA–TF–gene regulatory network in HCC. (**A**) Hierarchical clustering of differentially expressed genes (DEGs) expression profiling among samples. The color

gradient from blue to red indicates the scaled expression (Z score) from low to high. (**B**) Hierarchical clustering of differentially expressed miRNAs (DEMs) expression profiling among samples. (**C**) KEGG pathway enrichment analysis result for targets of DEMs. Scale from lightblue to darkblue indicates the -log *P* (log10 scaled) from low to high. (**D**) miRNA–TF–gene regulatory network for hub nodes. TFs, miRNAs and their target gene(s) are indicated in triangles, rounded rectangles and circles, respectively. Genes up-regulated and down-regulated in all patients are indicated in red and blue, respectively. Purple edges indicated TF–target regulation, while red edges indicated miRNA–target regulation. (**E**) DNA methylation level of *FOXO1* and *CPEB3* in whole gene region (up) and promoter region (down) between tumor and adjacent tissues. *P* was calculated by Student's *t*-test. (**F**) Copy number variation of *FOXO1* and *CPEB3* from GSCALite database.

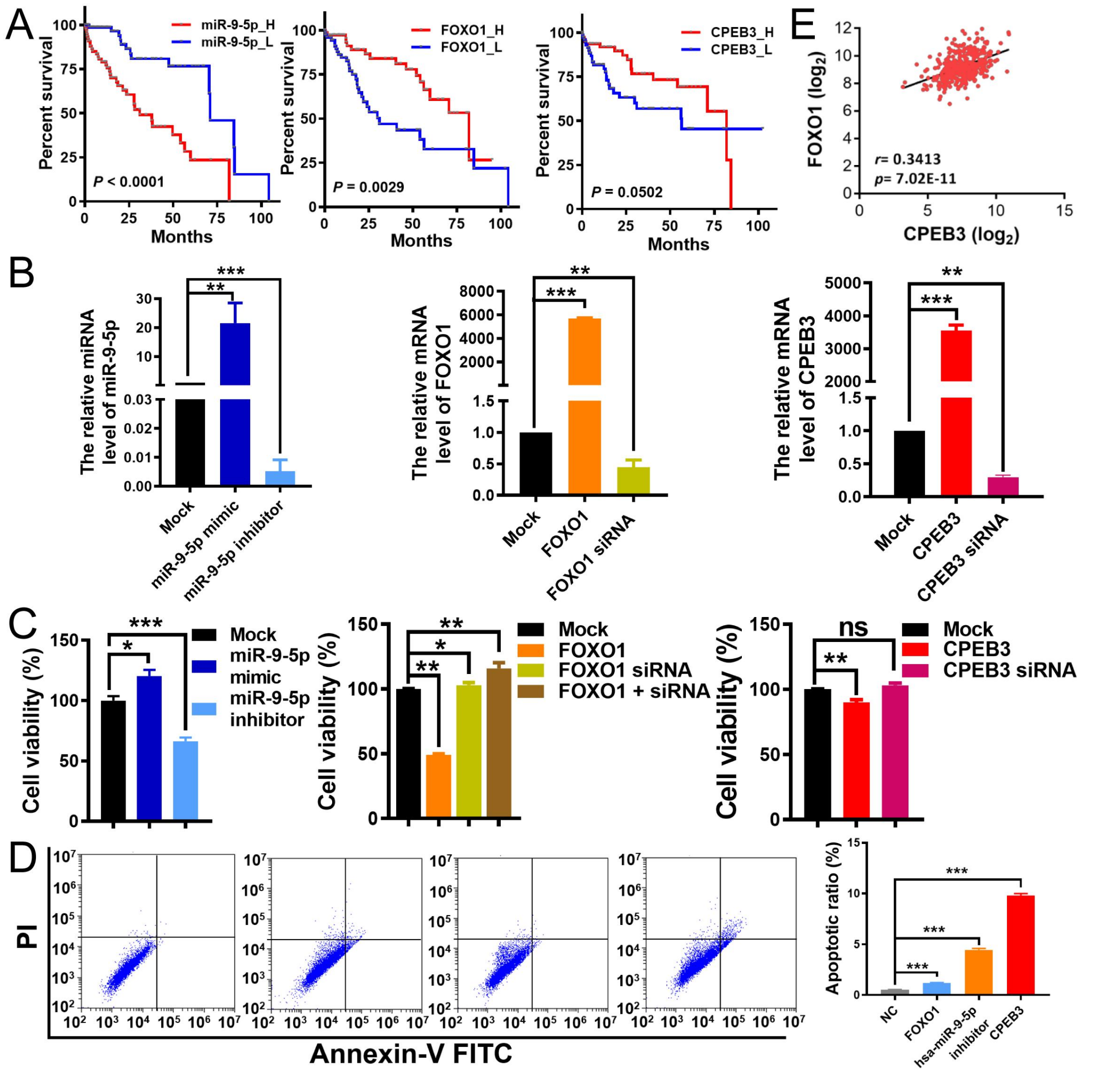

Figure S2. miR-9-5p promotes the growth of HCC cells *in vitro* and FOXO1, CPEB3 inhibit cell growth. (**A**) Kaplan-Meier survival analysis result of miR-9-5p, *FOXO1*, and *CPEB3* at 100 months (log-rank test). (**B**) RT-PCR showing transcriptional levels of the miR-9-5p, *FOXO1*, *CPEB3* after transfection with miR-9-5p mimic or its inhibitor (left), *FOXO1* or siFOXO1 (middle), and *CPEB3* or siCPEB3 (right) in Bel7402 cells, separately. Relative expression levels for treatment groups were calculated compared to the Mock (control group, normalized to 1). One-way ANOVA and Dunnett's multiple comparison test, Mean ± s.e.m., n = 3/group. (**C**) Cell viability of HepG2 cells after transfection with Mock (control cells group), miR-9-5p mimics, inhibitor as indicated (left); Transfected with Mock, *FOXO1* siRNA or *FOXO1* vector with or without siRNA (middle) and transfected with Mock, *CPEB3* siRNA or *CPEB3* vector with or without siRNA (right). Cell viability in treatment groups was calculated as relative values to Mock (normalized to 1). Non-parametric one-way ANOVA with Dunnett's multiple comparison post hoc test was performed. Mean ± s.e.m., n = 4/group. (**D**) Apoptosis induced by miR-9-5p inhibitor, FOXO1, and CPEB3 is detected using flow cytometry in HepG2 cells. A significant test was performed by Student's *t*-test. Mean ± s.e.m., n = 3/group. (**E**) Correlations between *FOXO1* and *CPEB3* expression levels were calculated by Pearson's correlation analysis based on all samples of TCGA HCC data. ∗ P < 0.05, ∗∗ P < 0.01, ∗∗∗ P < 0.001, ns: non-significant result.

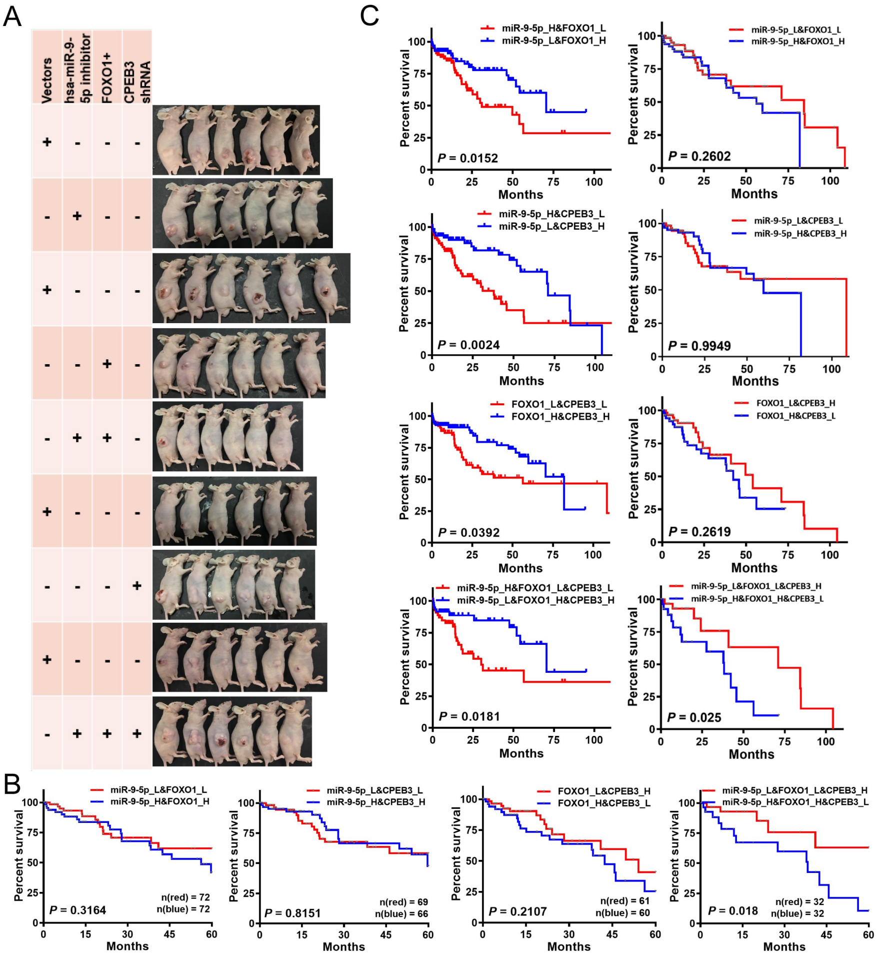

Figure S3. Effects of miR-9-5p/FOXO1/CPEB3 FFL *in vivo*. (**A**) Xenograft mouse models with different conducts of miR-9-5p, FOXO1 and CPEB3. (**B**) Kaplan-Meier survival analysis of combinations of two or three genes among low expressed miR-9-5p, low expressed *FOXO1* and high expressed *CPEB3* at 60 months (log-rank test) in HCC patients. (**C**) Results of Kaplan-Meier survival analysis of two or three genes' combinations in miR-9-5p/FOXO1/CPEB3 FFL at 100 months (log-rank test). L: Low-expression samples, H: High-expression samples.

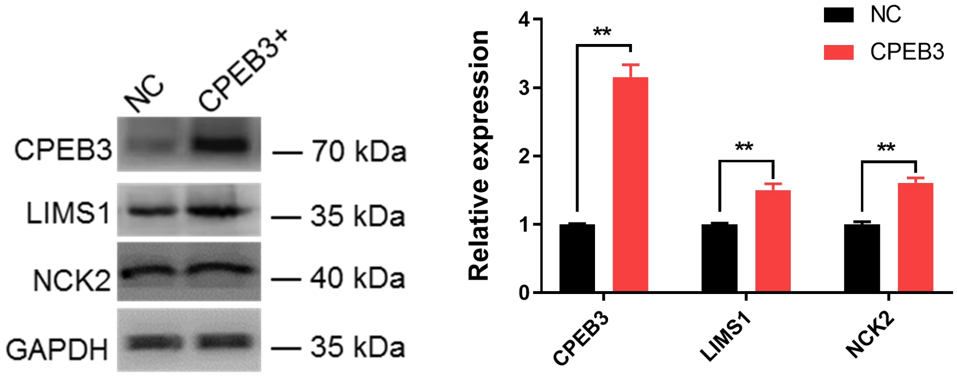

Figure S4. Potential downstream genes of CPEB3. Western blot analysis of CPEB3, LIMS1, NCK2 and GAPDH protein expression (left) and quantification (right) in HepG2 cells. Student's *t*-test. Mean ± s.e.m., n = 3/group. ** P < 0.01.

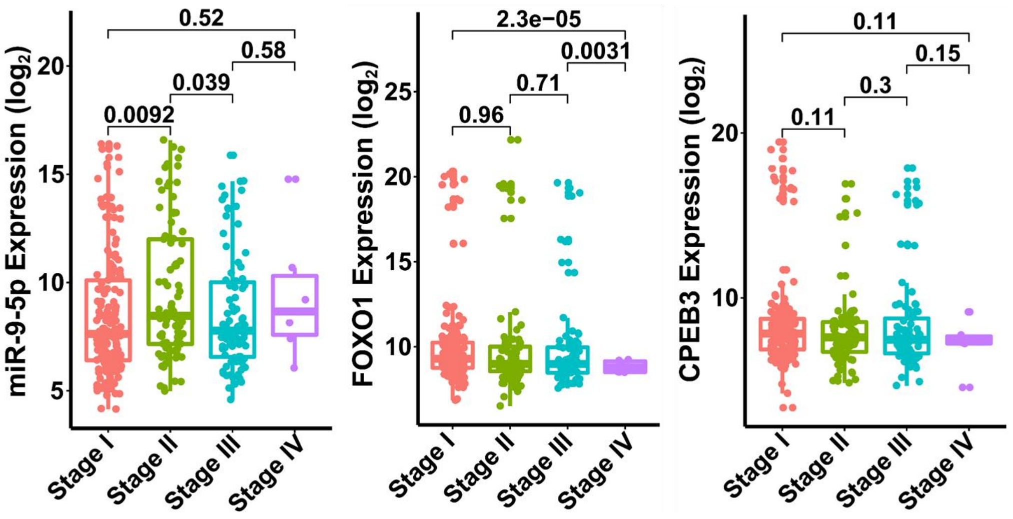

Figure S5. Gene expression in different pathological stages. The box plot demonstrates the relationship between miR-9-5p (left), *FOXO1* (middle) and *CPEB3* (right) gene expression (log2 RSEM (TCGA normalized mRNA expression)) and different pathological stages. Disease stage classification is derived from the American Joint Committee on Cancer Code, provided by TCGA database. Student's *t*-test was used.

2.2. Supplementary Tables

Table S1. Read statistics and quality control of RNA-seq. This table shows read counts and quality control of RNA-Seq sequencing data for *FOXO1* overexpression and controls.

**Sample**	**Reads count(paired)**	**GC%**	**q20%**	**q30%**	**Read length (bp)**	**Clean reads(Paired)**	**Mapping rate%**
HepG2-*FOXO1*	62108637	48.5	98.00	94.56	150	62087977	97.83
HepG2-NC	52526953	48	97.92	94.39	150	52509488	97.83

Table S3. Sequences of primers for real-time qPCR and oligonucleotides in this study.

**Primer function and name**	**Direction**	**Sequence**
**Real-time qPCR**		
Homo *GAPDH*	**F**orward primer	TCAACGACCACTTTGTCAAGCTCA
	**R**everse primer	GCTGGTGGTCCAGGGGTCTTACT
Homo *FOXO1*	**F**orward primer	CAGAACTGCTGGTGCCAGAATT
	**R**everse primer	GAGATTTCCCGCTCTTGCCAC
Homo *CPEB3*	**F**orward primer	TGGATGGTTCTCAGCCTTTGGA
	**R**everse primer	GCATAGCAGACACCACCGTACA
hsa-U6		CTCGCTTCGGCAGCACA
miR-9-5p		TCTTTGGTTATCTAGCTGTATGA
**Oligonucleotides**		
miR-9-5p mimics	sense (5'-3')	UCUUUGGUUAUCUAGCUGUAUGA
	antisense (5'-3')	AUACAGCUAGAUAACCAAAGAUU
Negative control (NC mimic)	sense (5'-3')	UUCUCCGAACGUGUCACGUTT
	antisense (5'-3')	ACGUGACACGUUCGGAGAATT
miR-9-5p inhibitor		UCAUACAGCUAGAUAACCAAAGA
Inhibitor NC		CAGUACUUUUGUGUAGUACAA
CPEB3 siRNA	sense (5'-3')	AAUAAUACCAUUUUAAUAGCA
	antisense (5'-3')	CUAUUAAAAUGGUAUUAUUAA
FOXO1 siRNA	sense (5'-3')	AGUUAAAAUCCAAUGUAUCUC
	antisense (5'-3')	GAUACAUUGGAUUUUAACUUU
Negative control (NC siRNA)	sense (5'-3')	UUCUCCGAACGUGUCACGUTT
	antisense (5'-3')	ACGUGACACGUUCGGAGAATT

Table S4. Clinical characteristics of 368 patients of liver hepatocellular carcinoma from TCGA.

**Neoplasm Disease Stage American Joint Committee on Cancer Code**	**Patients count**	**Percentage**
Stage I	172	46.74%
Stage II	85	23.10%
Stage III	3	0.82%
Stage IIIA	63	17.12%
Stage IIIB	8	2.17%
Stage IIIC	9	2.45%
Stage IV	3	0.82%
Stage IVA	1	0.27%
Stage IVB	2	0.54%
Not Available	22	5.98%
Sum	368	
**Viral Hepatitis Serology**	**Patients count**	**Percentage**
HBV	62	16.85%
HCV	19	5.16%
HBV or HCV	84	22.83%
Not Available	74	20.11%
UNKNOWN	129	35.05%
Sum	368	
**Vital Status**	**Patients count**	**Percentage**
Alive	277	75.27%
Dead	91	24.73%
Sum	368	
**Radiation Treatment Adjuvant**	**Patients count**	**Percentage**
YES	4	1.09%
NO	238	64.67%
Not Available	95	25.82%
UNKNOWN	31	8.42%
Sum	368	
**Pharmaceutical Adjuvant**	**Patients count**	**Percentage**
YES	14	3.80%
NO	222	60.33%
Not Available	95	25.82%
UNKNOWN	37	10.05%
Sum	368	
**Ablation Embolization Adjuvant**	**Patients count**	**Percentage**
YES	11	2.99%
NO	229	62.23%
Not Available	95	25.82%
UNKNOWN	33	8.97%
Sum	368	
